# Synthesis of Substituted α-Trifluoromethyl Piperidinic Derivatives

**DOI:** 10.3390/molecules22030483

**Published:** 2017-03-19

**Authors:** Sarah Rioton, Domingo Gomez Pardo, Janine Cossy

**Affiliations:** Laboratoire de Chimie Organique, Institute of Chemistry, Biology and Innovation (CBI), CNRS UMR 8231, ESPCI Paris, PSL Research University, 10 rue Vauquelin, 75231 Paris CEDEX 05, France; sarah.rioton@espci.fr (S.R.); domingo.gomez-pardo@espci.fr (D.G.P.)

**Keywords:** nitrogen heterocycles, fluorine, piperidine, trifluoromethyl group, ring expansion, cyclization, cycloaddition

## Abstract

A comprehensive survey of pathways leading to the generation of α-trifluoromethyl monocyclic piperidinic derivatives is provided (65 references). These compounds have been synthesized either from 6-membered rings e.g., pipecolic acid or lactam derivatives by introduction a trifluoromethyl group, from pyridine or pyridinone derivatives by reduction, and from 5-membered rings e.g., prolinol derivatives by ring expansion, from linear amines by cyclization or from dienes/dienophiles by [4 + 2]-cycloaddition.

## 1. Introduction

Functionalized piperidinic derivatives are among the most ubiquitous heterocyclic cores in natural products and bioactive compounds, therefore a huge number of methods has been developed to prepare piperidinic derivatives [1,2,3,4,5,6,7]. With respect to the biologically active targets, there is great interest in introducing substituents that can increase their biological activity which can be related to an increase of the lipophilicity, the bioavailability and the metabolic stability. In this context, the trifluoromethyl group is often used as a bioisostere of a chloride or a methyl group to modulate the steric and electronic properties of a lead compound or to protect a reactive methyl group from metabolic oxidation. This substituent can also increase the lipophilicity of molecules [8]. 

**Scheme 1:**
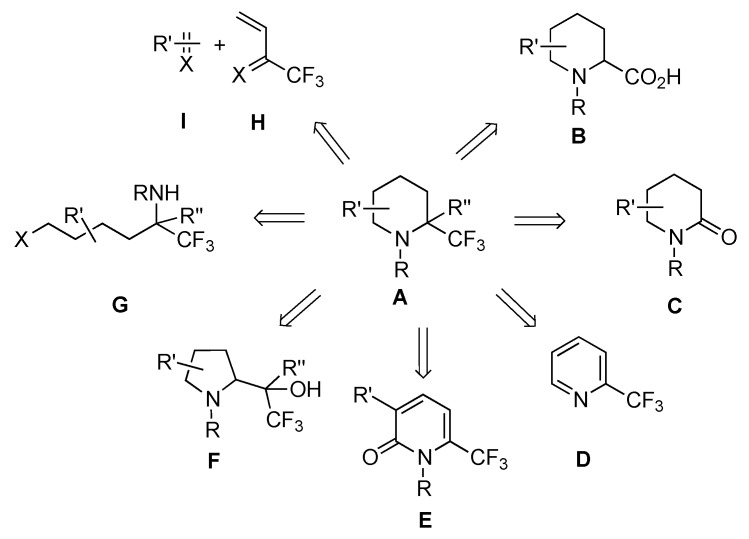
Precursors of α-trifluoromethyl piperidinic derivatives.

Herein, we report the synthesis of α-trifluoromethylpiperidinic derivatives of type **A**. These compounds have been synthesized either from 6-membered rings **B** and **C** by introduction of a CF_3_ group, from pyridines **D** or pyridinones **E** by reduction, from 5-membered rings **F** by ring expansion, from linear amines **G** by cyclization, from dienes/dienophiles **H**/**I** by cycloaddition (Scheme 1). We will only report the formation of monocyclic piperidinic derivatives, and of bicyclic derivatives only when they were transformed into the monocyclic derivatives.

## 2. From Cyclic Substrates

### 2.1. From 6-Membered Rings

The synthesis of α-trifluoromethylpiperidines **A** has been achieved from pipecolic acid, from δ-lactams, from pyridines and from pyridinones.

#### 2.1.1. From Pipecolic Acid

**Scheme 2:**
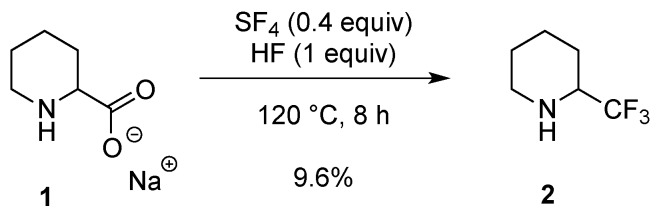
Synthesis of 2-trifluoromethyl piperidine from pipecolic acid.

The first synthesis of 2-(trifluoromethyl)piperidine (**2**) was realized in 1962 by Raash [9], from the sodium salt of pipecolic acid (**1**) that was treated with sulfur tetrafluoride (SF_4_) in the presence of HF at 120 °C. However, **2** was isolated in a very low yield of 9.6% (Scheme 2).

#### 2.1.2. From 2-Trifluoromethylpyridine

**Scheme 3:**
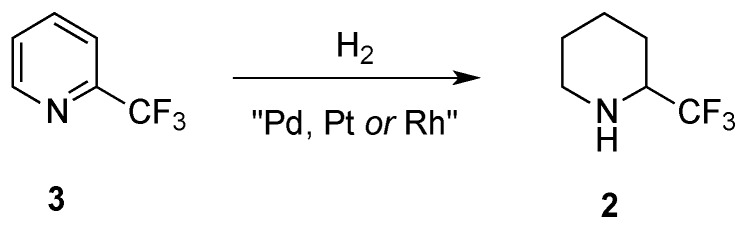
Hydrogenation of α-trifluoromethylpyridine.

One easy access to 2-trifluoromethylpiperidine (**2**) was realized by hydrogenation of the commercially available 2-trifluoromethylpyridine (**3**) in the presence of Pd, Pt or Rh catalysts (Scheme 3) [10,11,12].

#### 2.1.3. From Trifluoromethyl Pyridinones 

**Scheme 4:**
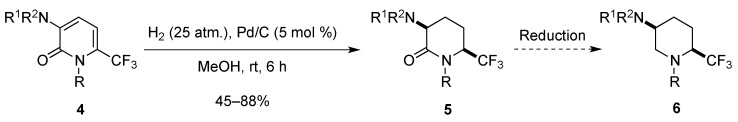
Hydrogenation of trifluoromethylpyridinones.

Access to trifluoromethylpiperidinones **5** from trifluoromethylpyridinones **4** has been developed by hydrogenation in the presence of Pd/C. Lactams **5** were isolated in 45%–88% yield and subsequent reduction should lead to the 5-amino 2-trifluoromethylpiperidines **6** (Scheme 4) [13].

#### 2.1.4. From δ-Lactams

**Scheme 5:**
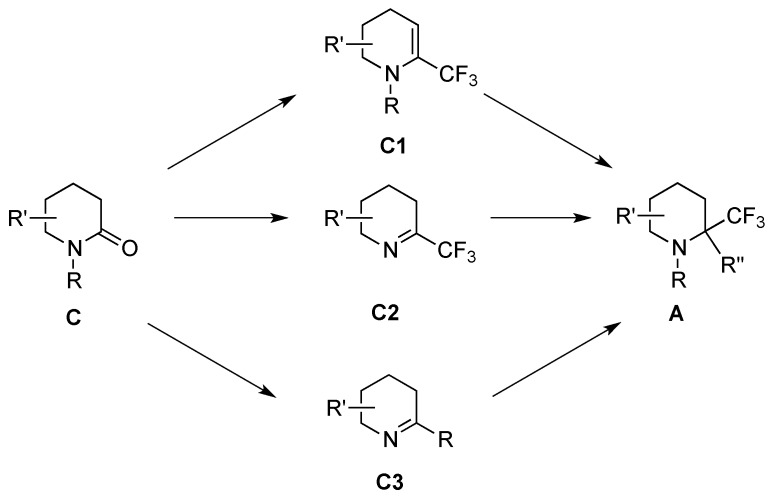
Synthesis of α-trifluoromethylpiperidines from δ-lactams.

Since the first access to 2-trifluoromethylpiperidine (**2**) different methods have been developed to produce α-trifluoromethylpiperidines of type **A** from δ-lactams **C** via either enamines **C1**, or imines **C2** or **C3** (Scheme 5).

##### From Enamines **C1**

**Scheme 6:**
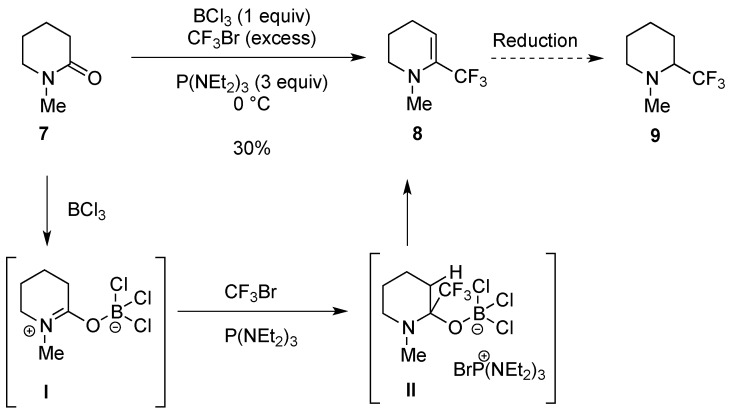
Synthesis of α-trifluoromethylpiperidine from δ-lactam via enamine.

α-Trifluoromethyl cyclic enamines **C1** can be good precursors of **A** as they are easily obtained from δ-lactams. When lactam **7** was treated with trichloroborane in the presence of an excess of CF_3_Br and HEPT, enamine **8** was formed in 30% yield via the formation of iminium species **I** which can be attacked by the CF_3_^–^ anion, generated from CF_3_Br. Intermediate **II** was thus produced and after a β-elimination, enamine **8** was formed (Scheme 6) [14]. A reduction of this enamine should lead to 2-trifluoromethylpiperidine **9**. It is worth noting that CF_3_Br is not easy to handle as it is a gas and alternatives to synthesize 2-trifluoromethylpiperidines from δ-lactams from imines **C2** and **C3** have been devised.

##### From Imines **C2**

Imines of type **C2** were synthesized in four steps from *N*-(diethoxymethyl)piperidin-2-one (**10**). First, **10** was subjected to a Claisen condensation with ethyl trifluoroacetate (NaH, CF_3_CO_2_Et) and, after deprotection under acidicic conditions (HCl), the corresponding fluorinated acyl lactam **11** was isolated as the hydrate, due to the ability of the electron-withdrawing CF_3_ group to stabilize the tetrahedral adducts of such type of compounds [15].

**Scheme 7:**
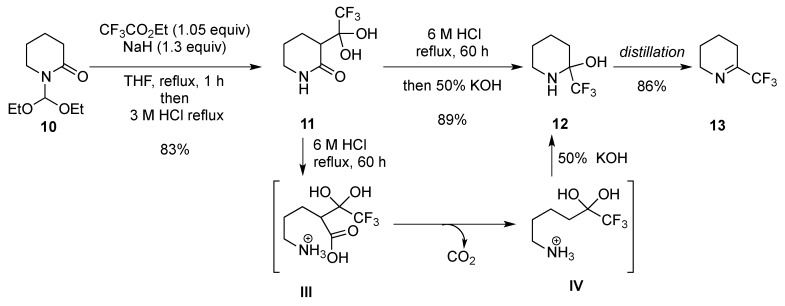
Synthesis of α-trifluoromethylpiperidine from δ-lactam via imine.

The hydrolysis and the decarboxylation of perfluoro acyl lactam **11** under acidic conditions (6 M HCl) and heating at reflux led to **III**. When the decarboxylation was complete, the reaction was carefully made alkaline with KOH and hemiaminal **12** was obtained. After azeotropic removal of water or distillation, **12** was transformed into imine **13** (Scheme 7) [15].

**Scheme 8:**
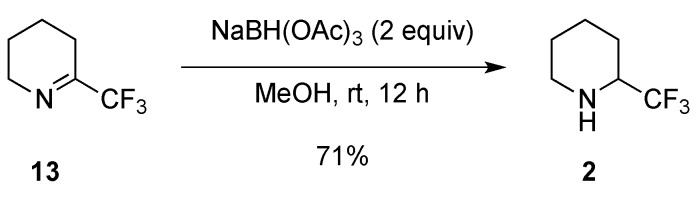
Synthesis of α-trifluoromethylpiperidine from an imine by reduction.

Imine **13** can be involved in a variety of reactions such as reduction, phosphorylation, alkylation under Friedel-Craft conditions, and Ugi-type reactions, producing a diversity of α-trifluoromethyl piperidinic derivatives. Imine **13** was transformed to 2-trifluoromethylpiperidine (**2**) in a yield of 71% by reduction with NaBH(OAc)_3_ in MeOH (Scheme 8) [15].

**Scheme 9:**
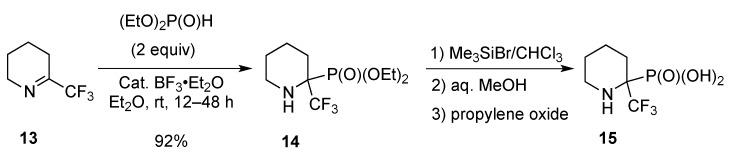
Synthesis of 2-(trifluoromethyl)-2-ethylphosphonate piperidine from imine.

When **13** was treated with diethyl phosphite in the presence of boron trifluoride etherate (BF_3_**^.^**OEt_2_) as the catalyst, 2-(trifluoromethyl)-2-ethylphosphonate piperidine (**14**) was obtained (92%) and easily transformed to the corresponding α-trifluoromethyl substituted cyclic α-aminophosphonic acid **15** by treatment with trimethylbromosilane in CHCl_3_ followed by the addition of aqueous MeOH. It is worth pointing out that to avoid the formation of the ammonium salt, due to the liberation of HBr during the hydrolysis of the ester, the addition of propylene oxide was necessary and the free amino acid **15** was isolated (Scheme 9) [16].

**Scheme 10:**
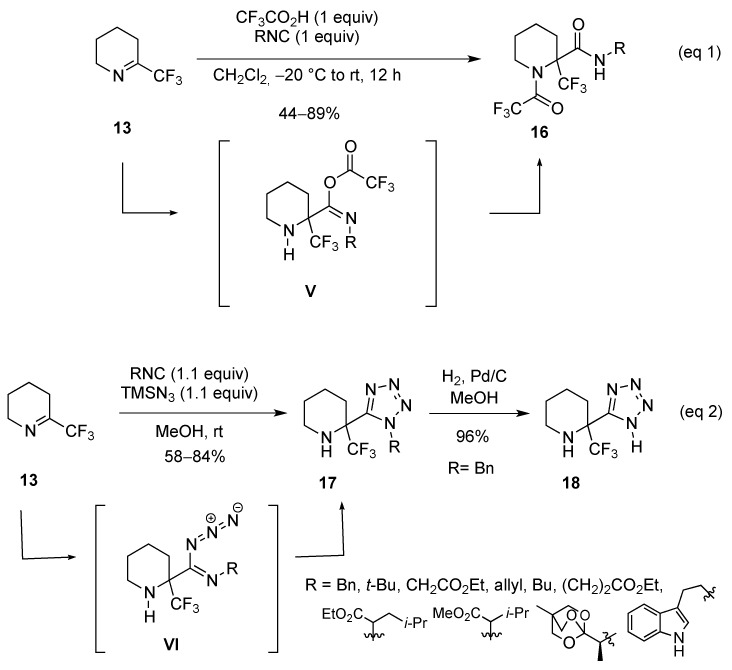
Synthesis of α-trifluoromethylpiperidines by Ugi-type reactions.

Imine **13** was transformed to the piperidinic derivatives **16** when subjected to an Ugi-type reaction using isocyanides. Thus, when **13** was treated with an isocyanide (RNC) in the presence of CF_3_CO_2_H, piperidine **16** was formed via intermediate **V** (Scheme 10, eq. 1) [17]. It is worth mentioning that an azido-Ugi reaction was performed using isocyanate (RNC) in the presence of TMSN_3_ in MeOH. Under these conditions, tetrazole **17** was obtained via intermediate **VI**. In addition, when benzylisocyanate (BnNC) was used, the corresponding piperidyltetrazole (**17**, R = Bn) was isolated and debenzylated by hydrogenolysis (H_2_, Pd/C, MeOH) to produce **18** in good yield (96%) (Scheme 10, eq. 2) [18].

**Scheme 11:**
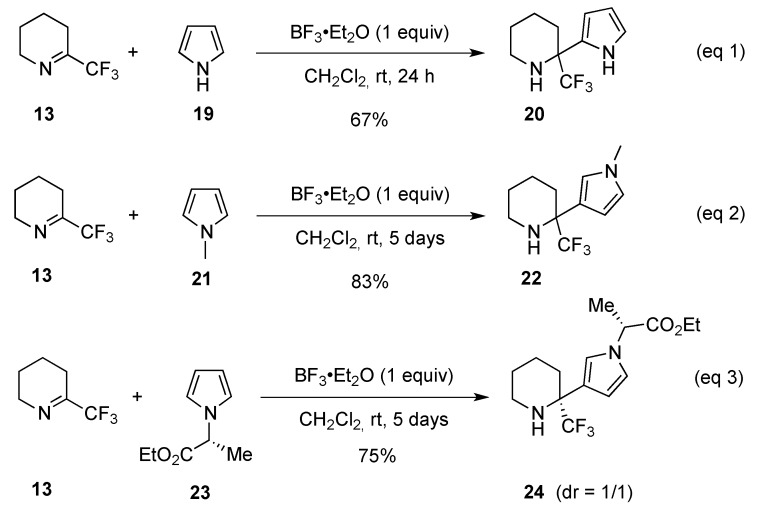
Synthesis of α-trifluoromethylpiperidines by Friedel-Craft reactions with pyrroles.

If tetrazole can be introduced at the α-position of α-trifluoromethylpiperidyl derivatives, using an Ugi-type reaction, other heterocycles such as pyrroles, indoles can also be introduced in the α-position by using a Friedel-Craft reaction. When imine **13** was treated with the non-protected pyrrole **19** in the presence of BF_3_**^.^**OEt_2_, the unexpected piperidine **20**, possessing a C2-substituted pyrrole was obtained (67%) (Scheme 11, eq. 1). On the contrary, when the *N*-methylpyrrole **21** was involved in the Friedel-Craft reaction, piperidine **22**, substituted in the α-position by the C3-substituted pyrrole, was isolated (Scheme 11, eq. 2). The same regioselectivity at C3 was observed with pyrrole **23**. It is worth mentioning that no diastereoselectivity was observed with pyrrole **23** as a ratio of 1 to 1 was observed for **24** (Scheme 11, eq. 3). As noticed, the reaction with non-protected and protected pyrroles is very regioselective. This regioselectivity can be the result of steric interactions between the *N*-substituent of the pyrrole and the cyclic imine which can be quite significant due to the bulkiness of the trifluoromethyl group and its specific nature and, the C3 isomers were formed. When less steric interactions were present, e.g., with unprotected pyrroles, the C2-isomers were formed (Scheme 11, eq. 1) [19,20].

**Scheme 12:**
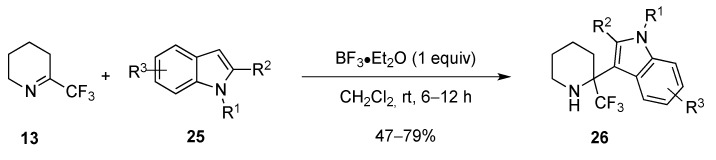
Synthesis of α-trifluoromethylpiperidines by a Friedel-Craft reaction with indoles.

Indoles such **25** can also be involved in a Friedel-Craft reaction with imine **13** leading to the piperidines **26** in good yield (Scheme 12) [21].

##### From Imines **C3**

**Scheme 13:**
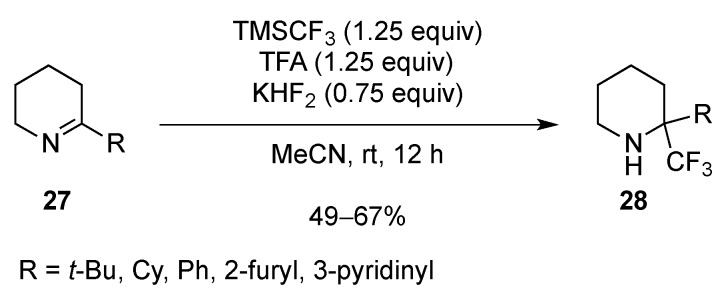
Synthesis of α,α-disubstituted piperidines.

In imines **13**, the CF_3_ group is initially located in the α-position and then different R substituents can also be introduced in the α-position. From an imine intermediate, the reverse strategy can be used to access compound **A**, e.g., imines are synthesized from a δ-lactam and then the trifluoromethyl group is introduced. Thus, when imines **27**, prepared from the corresponding δ-lactams, were treated with the Ruppert-Prakash reagent (TMSCF_3_) in the presence of TFA and KHF_2_, in MeCN, the α,α-disubstituted piperidines **28** were isolated in good yields. The activation of imines **27** under acidic conditions was necessary to obtained piperidines **28** (Scheme 13) [22].

It is worth mentioning that all the 2-trifluoromethylpiperidines were obtained in a racemic form, however, recently, we have reported a method that allows the synthesis of optically active substituted 2-trifluoromethylpiperidines by using a stereospecific ring expansion applied to 2′-trifluoromethylprolinols of type **F**.

### 2.2. From 5-Membered Rings

The ring expansion of pyrrolidines to piperidines via an aziridinium intermediate under kinetic and thermodynamic conditions is well-established [23,24,25,26,27,28]. This method was used to access piperidines **30**, **30′** from prolinols **29**, **29′**.

**Scheme 14:**
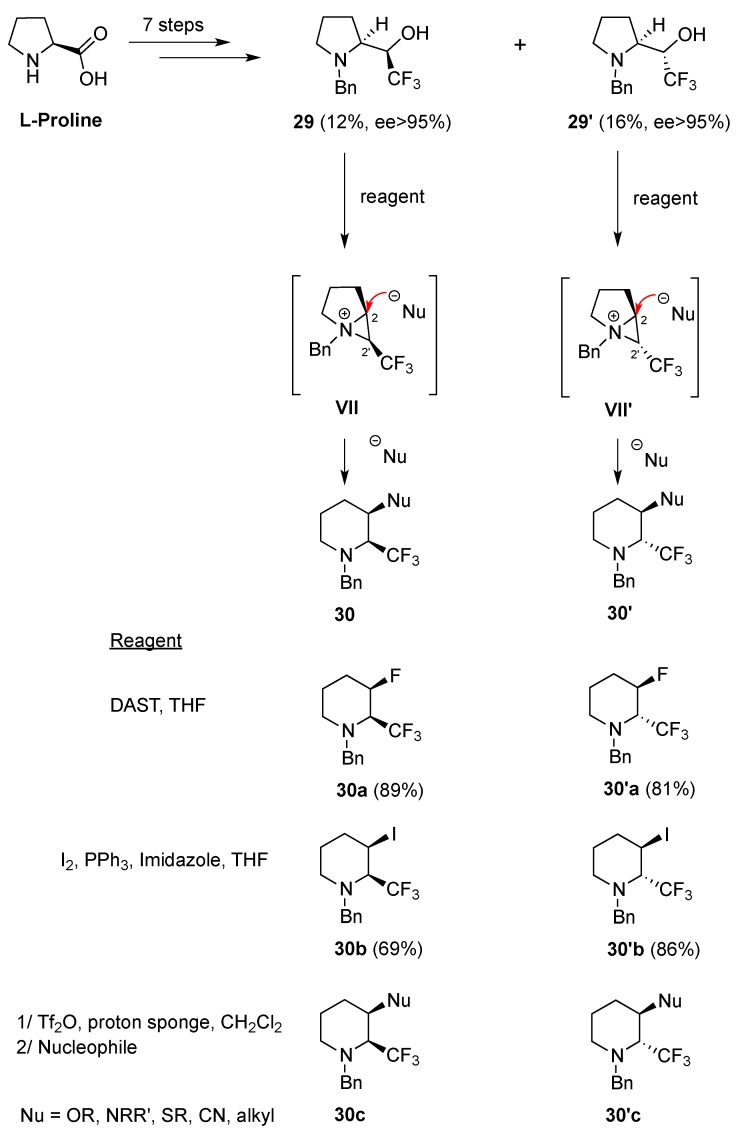
Synthesis of C3-substituted α-trifluoromethylpiperidines from l-proline.

Prolinols **29** and **29′** were prepared from l-proline in a seven-step sequence [29]. When prolinols **29** and **29′** were treated with DAST, I_2_/PPh_3_ or by Tf_2_O, followed by the addition of different nucleophiles (alcohols, amines, thiols, cuprates, cyanides), a diversity of 2-(trifluoromethyl)- 3-substituted piperidines **30** and **30′** were obtained with good diastereo- and enantioselectivities (de > 94% and ee > 99%). The regioselective attack of the nucleophiles on the aziridinium intermediates **VII**/**VII′** is due to the presence of the CF_3_ group (Scheme 14) [29,30]. 

## 3. From Non-cyclic Substrates

To synthesize 2-trifluoromethyl-substituted piperidines from non-cyclic substrates, a cyclisation step or a cycloaddition is necessary to access the piperidine skeleton.

### 3.1. Cyclization

#### 3.1.1. By Metathesis

**Figure 1:**
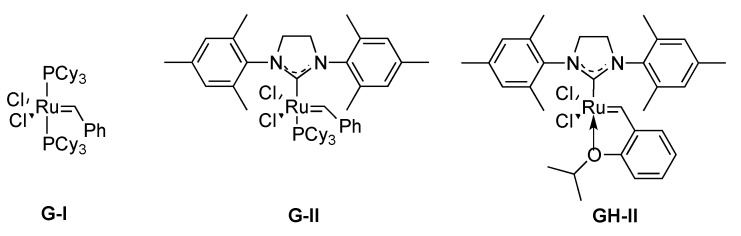
Catalysts for olefin metathesis.

Metathesis reactions have changed the way molecules are constructed due to the commercialization of robust catalysts such as the Grubbs first and second generation catalysts, **G-I** and **G-II**, and the Grubbs-Hoveyda catalysts **GH-II** (Figure 1). Metathesis is a powerful method to access both carbo- and heterocyclic ring systems [31,32,33,34,35] and the reaction is useful to synthesize piperidines and particularly to synthesize 2-trifluoromethylpiperidinic derivatives.

##### From Dienes

**Scheme 15:**
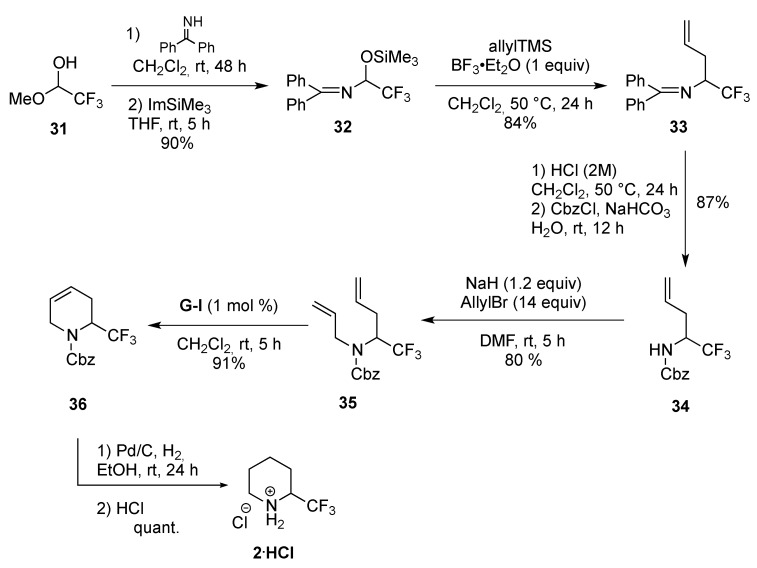
Synthesis of α-trifluoromethylpiperidine from trifluoromethylhemiacetal.

The ammonium salt of the 2-trifluoromethylpiperidine **2^.^**HCl was formed from **36**, which was obtained from the α-trifluoroaminodiene **35** when treated with **G-I**. This diene has been synthesized in six steps from the hemiacetal **31** which was transformed into imine **32** by treatment with the imine of benzophenone followed by a silylation step (ImSiMe_3_). After an allylation under acidic conditions (allylTMS, BF_3_**^.^**OEt_2_), followed by a deprotection/protection sequence, the allyltrifluoroamine **34** was isolated and allylated to produce the piperidinic core precursor **35**. Thus, after treatment of **35** with **G**-**I** catalyst (1 mol %) in dichloromethane at rt, the unsaturated piperidine **36** was isolated in 91% yield. After hydrogenation (Pd/C, EtOH, rt, 24 h) and treatment with HCl, **2^.^**HCl was isolated quantitatively (Scheme 15) [36,37]. Compared to the synthesis of **2** from pipecolic acid the synthesis of **2** from **31** is more efficient in terms of yield (47.9% versus 9.6%).

**Scheme 16:**
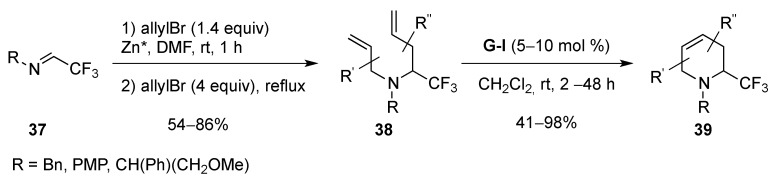
Synthesis of α-trifluoromethylpiperidines from trifluoromethylimines.

A shorter synthesis of unsaturated piperidines from imines **37** using a Barbier-type reaction (allylBr, Zn, DMF, rt) followed by a *N*-allylation has been reported. The resulting dienic amino compound **38** was isolated in good yied. After treatment with **G-I** (5–10 mol %) the unsaturated piperidines **39** were isolated (Scheme 16) [38].

**Scheme 17:**
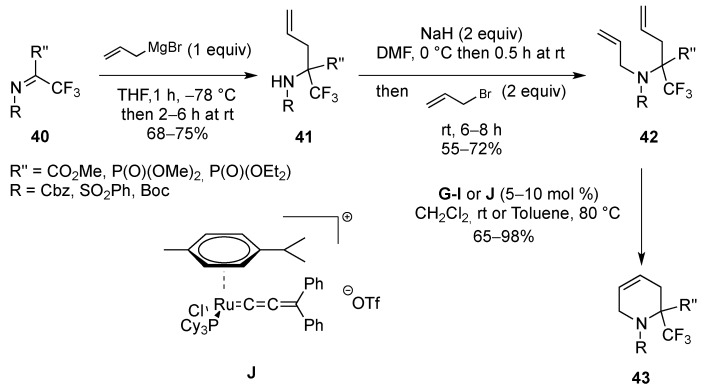
Synthesis of α,α-disubstituted unsaturated piperidines from trifluoromethylimines.

Starting from imines, the strategy is very versatile to access a variety of unsaturated piperidines and, particularly, 2-(trifluoromethyl)-2-substituted tetrahydropyridines. Thus, when imines **40** were treated with allylmagnesium bromide (THF, –78 °C) then allylated (NaH, allylbromide) dienes **42** were isolated in good yields and then involved in a ring-closing metathesis (RCM) using different catalysts such as **G-I** or the ruthenium catalyst **J** [39]. It is worth noting that the yields obtained with **G-I** are better than with catalyst **J** (Scheme 17) [40,41,42,43]. 

**Scheme 18:**
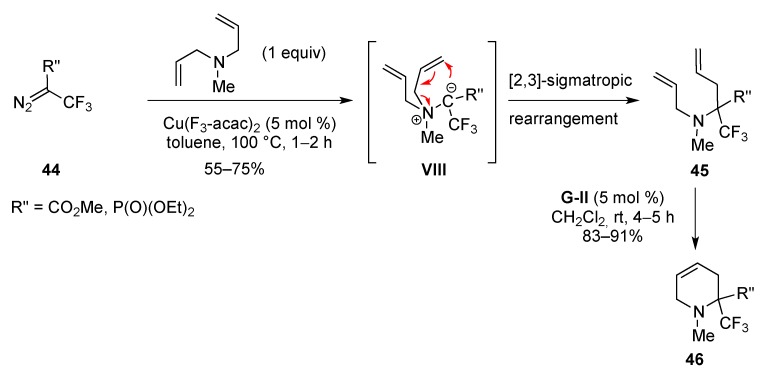
Synthesis of α,α-disubstituted piperidines from trifluorodiazo derivatives.

It is worth noting that the dienes, precursors of 2-(trifluoromethyl)-unsaturated piperidines, could be synthesized from trifluorodiazo derivatives **44**. Treatment of *N*,*N*-diallyl-*N*-methylamine by the diazo compound **44** in the presence of the copper catalyst [Cu(F_3_-acac)_2_], produced ylide **VIII** which, after a [2,3]-sigmatropic rearrangement, led to the dienic derivatives **45**, precursor of the unsaturated piperidines **46** (Scheme 18) [43]. 

**Scheme 19:**
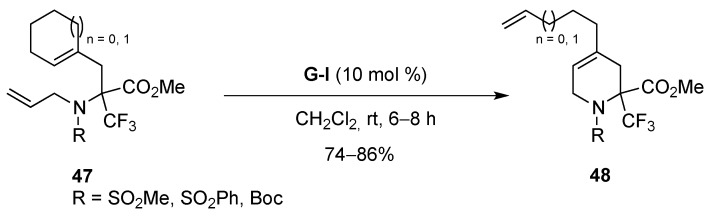
Synthesis of α-trifluoromethylpiperidines using a ROM/RCM sequence.

When the ring-opening metathesis (ROM)/ ring-closing metathesis (RCM) was applied to **47**, using the **G-I** catalyst (10 mol %), the unsaturated piperidines **48**, substituted at C4, were isolated in good yields (74%–86%) (Scheme 19) [44].

##### From Enynes

**Scheme 20:**
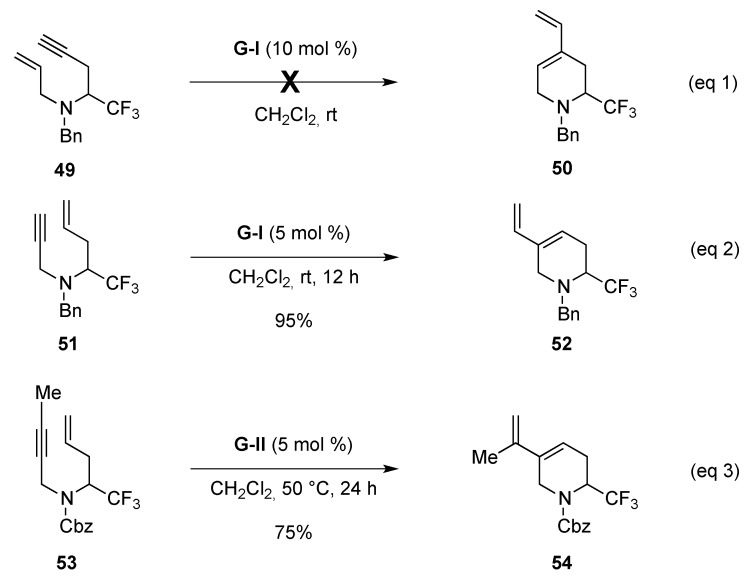

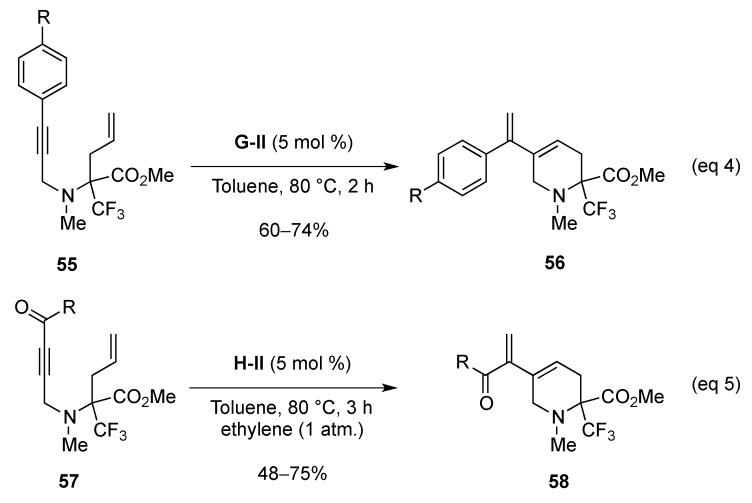
Synthesis of α-trifluoromethylpiperidines by a RCM to enynes.

It is worth noting that a substituent at C4 could not be introduced on the piperidinic core by using a RCM applied to enyne **49** (Scheme 20, eq. 1). On the contrary, 2-(trifluoromethyl) unsaturated piperidines substituted at C5 can be produced by applying a RCM to enyne **51**. When this latter was treated with **G-I** (5 mol %) the resulting unsaturated piperidine **52** was obtained with an excellent yield (95%) (Scheme 20, eq. 2). 

The alkyne can be substituted by different alkyl groups (compound **53**), aryl groups (compound **55**) and ketone (compound **57**). In all cases, the corresponding unsaturated piperidinic compounds substituted at C5, were isolated in good yields (Scheme 20, eqs. 3, 4 and 5) [37,38,45]. 

It is worth mentioning that in the case of **55**, when the aryl group is substituted in the *para* position the yields in **56** are good (60%–74%). On the contrary, when the aryl group is substituted in the *ortho* position, **55** is not transformed into **56** whatever the catalyst used, either the **G-II** or the **GH-II** catalysts [45].

#### 3.1.2. By Cycloaddition

Only the cycloadditions leading to the monocyclic piperidinic core will be reported thus, the [2 + 2]-cycloaddition of allenynes [46] and the Paulson-Khan reaction [38,43,47] will be excluded.

##### [4 + 2]-Cycloaddition: Aza Diels-Alder

If imines are used as dienophiles, activation of the imine moiety either by electron-withdrawing substituents or by a Lewis acid is required for a successful [4 + 2]-cycloaddition. Imines **59**, possessing three electron-withdrawing groups, are very reactive and the aza Diels-Alder reaction was achieved at low temperature in the presence of different dienes **60**, to produce the corresponding unsaturated piperidines **61** in good yields. It is worth mentioning that phosphonylimines were less reactive than sulfonylimines as the reaction has to be performed at higher temperature and longer reaction times were required (120 h for phosphonylimines versus 14 h for sulfonylimines) (Table 1) [48].

**Scheme 21:**
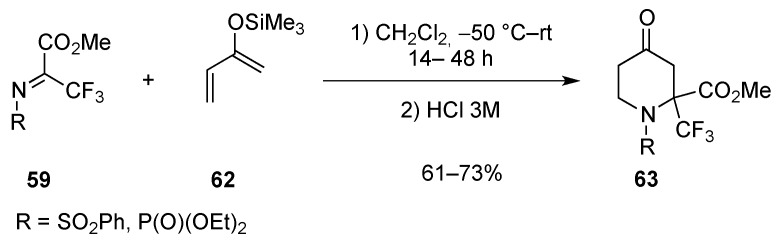
Synthesis of piperidinones by an aza Diels-Alder reaction.

When the silyloxydiene **62** was involved in the aza Diels-Alder reaction, piperidinone **63** was isolated after acidic treatment (Scheme 21) [48]

##### Aza Diels-Alder Reaction with 1-Azadiene

**Scheme 22:**
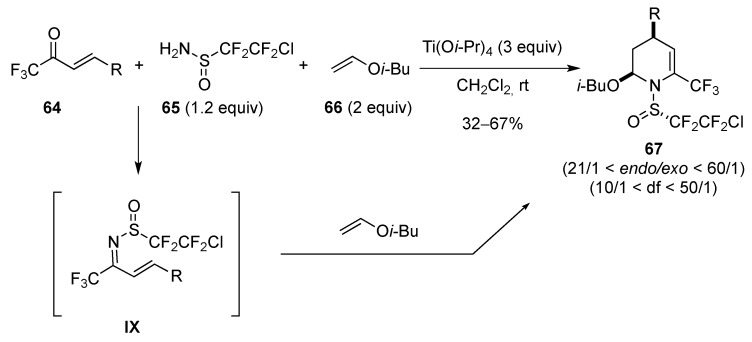
Aza Diels-Alder reaction with 1-azadienes.

The unsaturated piperidine **67** was obtained from an aza Diels-Alder reaction between the *iso*-butyl vinyl ether **66** and the sulfinimine intermediates **IX** generated *in situ* from the trifluoromethyl α,β-unsaturated ketone **64** and the sulfinamine **65**. In this aza Diels-Alder reaction, the HOMO orbital of **66** reacts with the LUMO orbital of the azadienes **IX**. The trifluoromethyl-piperidines **67** were obtained with a good diastereoselectivity in favor of the endo product. In addition, when the reaction was performed with an optically active sulfinamine, the cycloadduct was obtained with a good enantioselectivity (ee > 99%) (Scheme 22) [49].

#### 3.1.3. 1,4-Addition: Aza-Michael

**Scheme 23:**
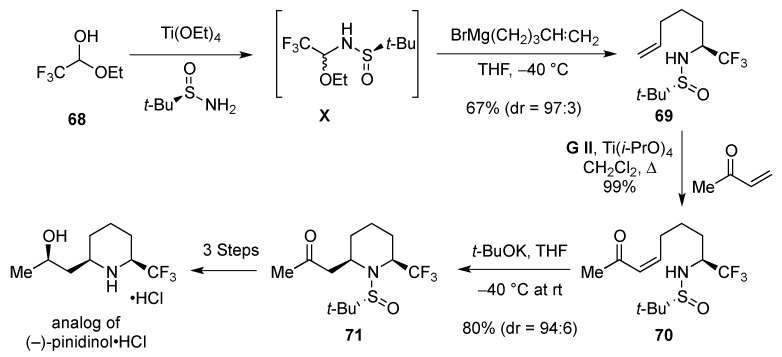
Synthesis of α-trifluoromethylpiperidine by aza-Michael addition.

A Michael acceptor, tethered to a nucleophilic amine, can undergo an intramolecular 1,4-addition with concomitant formation of a piperidinic core. After the preparation of the optically active ω-amino α,β-unsaturated ketone **70** in three steps from the trifluoromethylhemiacetal **68**, the aminoketone **70** was treated under basic conditions to produce the 2-trifluoromethyl-substituted piperidine **71** in 80% yield and with an excellent diastereoselectivity of 94:6 in favor of the *cis*-isomer. This piperidine was transformed to an analog of pinidinol (Scheme 23) [50].

**Scheme 24:**
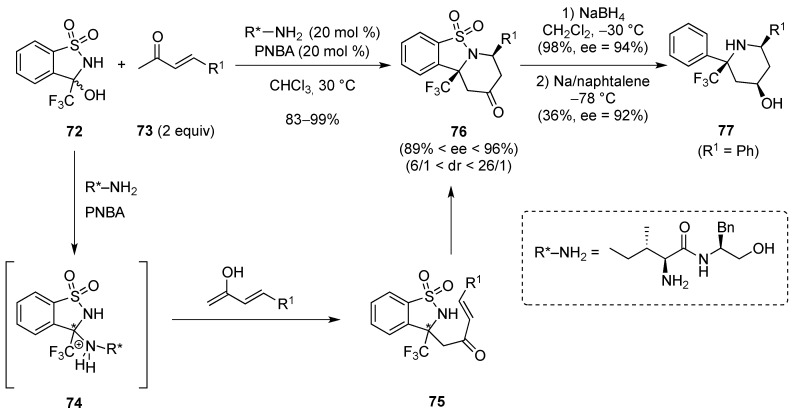
Synthesis of chiral α-trifluoromethylpiperidines.

2-Trifluoromethylpiperidines with a quaternary center at C2 were synthesized from hemiaminal **72** and an α,β-unsaturated ketone **73** in the presence of a chiral amine ((2*S*,3*S*)-2-amino-*N*-((*S*)-1-hydroxy-3-phenyl-propan-2-yl)-3-methyl-pentanamide) under acidic conditions [*para*-nitrobenzoic acid (PNBA)]. Hemiaminal **72** reacts with the chiral amine to form intermediate **74** which then reacts with the enolized form of **73** to produce intermediate **75**. An intramolecular aza-Michael reaction then occurs and the tricyclic compounds **76** were isolated in good yields and with excellent dr(s) and ee(s). After reduction of the ketone (NaBH_4_) and cleavage of the C–S and N–S bonds (sodium naphthalide), piperidine **77** (R^1^ = Ph) was isolated with a good enantiomeric excess (ee = 92%) (Scheme 24) [51].

#### 3.1.4. Electrophilic-Induced Cyclization

**Scheme 25:**
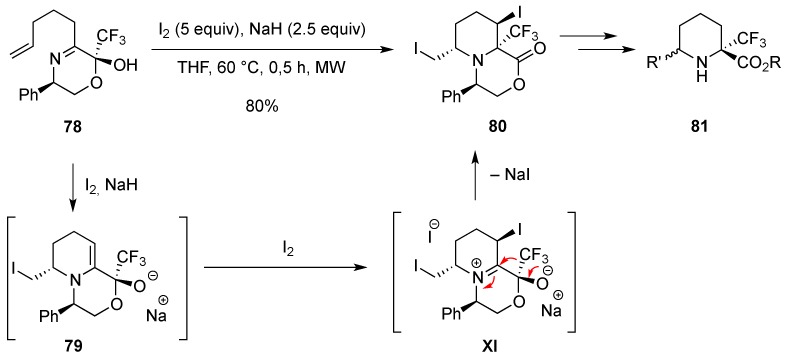
Synthesis of α-trifluoromethylpipecolic acid derivatives.

Amino-iodo cyclization has been used to prepare 2-trifluoromethylpipecolic acid derivatives starting from the ω-unsaturated imine **78**. The transformation of **79** to **80** was realized in a one-pot process with an excellent yield of 80%. After treatment of imine **78** with iodine in the presence of NaH, the bicyclic product **79** was formed and, when reacted with I_2_, a migration of the CF_3_ group took place leading to the diiodo derivative **80** via an iminium alcoholate intermediate **XI**. The bicyclic compound **80** was then transformed into a variety of 2-trifluoromethylpipecolic acid derivatives **81** in a few steps (Scheme 25) [52,53]. 

If in the electrophilic-induced cyclization of ω-aminoalkenes the CF_3_ group was present in the starting material, on the contrary, in the electrophilic-induced cyclization of ω-amino alkynes the CF_3_ was introduced after the cyclization.

**Scheme 26:**
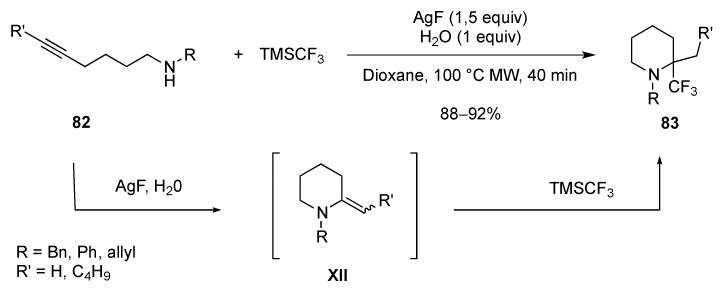
Synthesis of α-trifluoromethylpiperidines by electrophilic-induced cyclization.

When the ω-amino alkynes **82** were treated with AgF in the presence of H_2_O, a hydroamination takes place to produce the enamine intermediate **XII**, which was reacted with TMSCF_3_ to produce 2-trifluoromethylpiperidines **83** with excellent yields (88%–92%) (Scheme 26) [54].

#### 3.1.5. Nucleophilic Attack of Iminium Ions

**Scheme 27:**
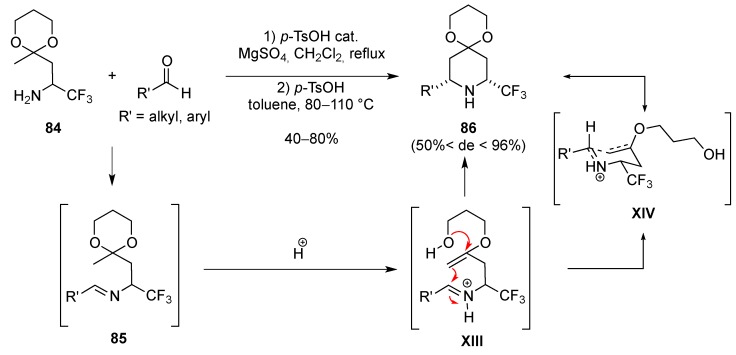
Synthesis of α-trifluoromethylpiperidines by an intramolecular Mannich reaction.

In reaction where iminium ions are involved, the iminium ion is attacked by a nucleophile such as in a Mannich type reaction and a Prins cyclization. A diastereoselective synthesis of 2-trifluoromethylpiperidines was realized by using an intramolecular Mannich reaction starting from the trifluoromethyl amine **84**. After condensation of the latter with aldehydes, the formed imines **85** were transformed to the iminium intermediates **XIII** under acidic conditions (*p*-TsOH, refluxing toluene). A cyclization is taking place to produce the 2-trifluoromethylpiperidinic derivatives **86**. The diastereoselectivity can be explained by the six-membered ring chair transition states **XIV**, in which the steric interactions are minimized (Scheme 27) [55,56,57].

**Scheme 28:**
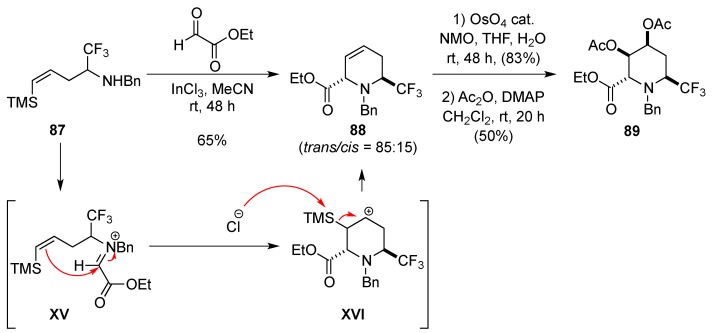
Synthesis of an α-trifluoromethylpiperidine by a silyl-aza-Prins reaction.

A silyl-aza-Prins reaction was also used to prepare highly functionalized 2-trifluoromethyl- piperidines. Treatment of the vinyl silyl trifluoromethyl amine **87** with ethyl glyoxylate in the presence of InCl_3_ led to **88** via the iminium intermediate **XV**. After an intramolecular attack of the iminium **XV** by the vinyl silane, intermediate **XVI** was formed, a desilylation took place and piperidine **88** was isolated in 65% yield. This piperidine can then be transformed to the highly functionalized 2-trifluoromethylpiperidine **89** (Scheme 28) [58].

#### 3.1.6. Intramolecular Condensation of Aminoketones and Aminoesters

**Scheme 29:**
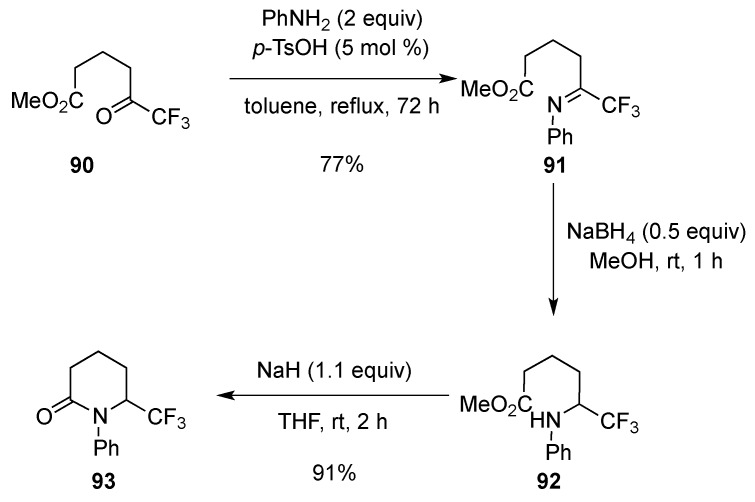
Synthesis of a trifluoromethyllactam from a ketoester.

Intramolecular lactamization and hemiaminalization were used to build up the C–N bond of the 2-trifluoromethylpiperidine derivatives. 2-Trifluoromethyllactam **93**, which can led to the corresponding piperidine by reduction, was synthesized in three steps from ketoester **90**. After treatment of **90** with aniline (*p*-TsOH, toluene, reflux), imine **91** was reduced to the δ-aminoester **92** which was cyclized to the corresponding lactam **93** under basic conditions (NaH, THF) (Scheme 29) [59].

**Scheme 30:**
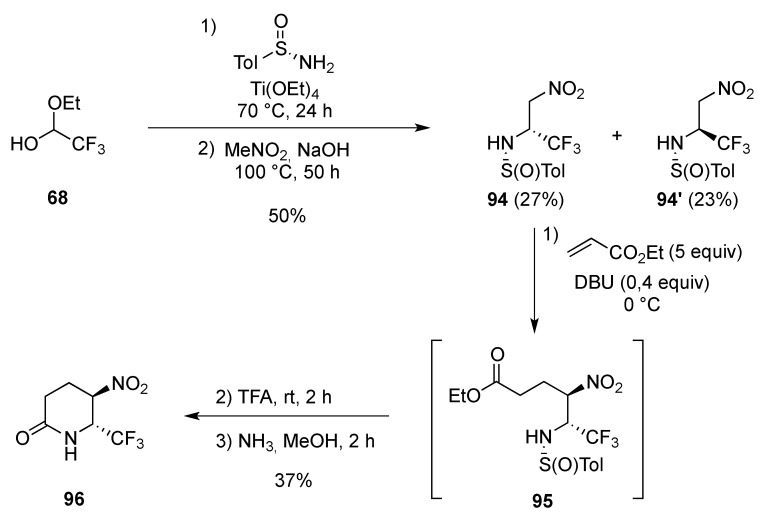
Synthesis of a trifluoromethyllactam from a trifluoromethylhemiacetal.

The α-substituted trifluoromethyl-δ-lactam **96** was prepared from the α-trifluoromethyl nitrosulfinamine **94**, prepared in two steps from trifluoromethylhemiacetal **68**. After a 1,4-addition of **94** to ethyl acrylate under basic conditions, the resulting aminoester **95** was treated with TFA to produce, after deprotection (NH_3_, MeOH), the trifluoromethyl-δ-lactam **96** (Scheme 30) [60].

**Scheme 31:**
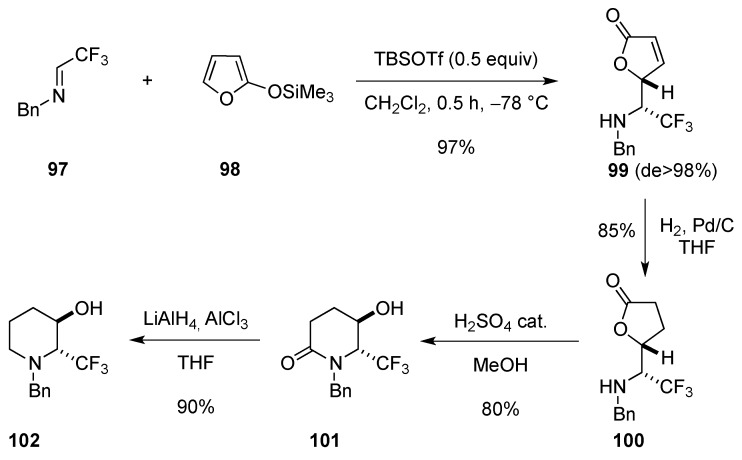
Synthesis of an α-trifluoromethylpiperidine by lactamization.

A lactamization was also utilized to access 2-trifluoromethyl-3-hydroxypiperidine **102**. The latter was synthesized from aldimine **97** and the 2-trimethylsilyloxyfuran **98**, and transformed into the unsaturated lactone **99** after treatment with TBSOTf. After hydrogenation, the obtained lactone **100** was transformed to the δ-lactam **101** under acidic conditions (H_2_SO_4_, 80%), and then to the 2,3-disubstituted piperidine **102** by reduction (LiAlH_4_, AlCl_3_) (Scheme 31) [61]. 

**Scheme 32:**
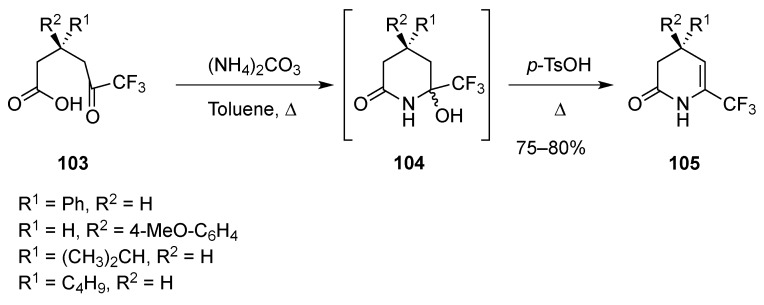
Synthesis of unsaturated lactams from carboxylic trifluoromethyl ketones.

Due to the high reactivity of trifluoromethyl ketones they can be attacked by weak nucleophiles such as amines to form a hemiaminal. When the carboxylic trifluoromethyl ketones **103** were treated with ammonium carbonate in refluxing toluene, the six-membered ring hemiaminal intermediates **104** were formed and dehydrated under acidic conditions (*p*-TsOH, reflux) to furnish the unsaturated lactams **105** (Scheme 32) [62].

**Scheme 33:**
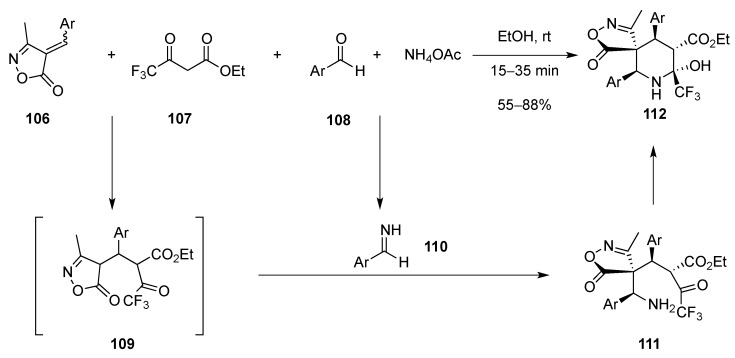
Synthesis of highly substituted α-trifluoromethylpiperidines.

Highly substituted 2-trifluoromethylpiperidines **112** were obtained from isoxazoles **106**, trifluoromethylketoester **107**, aldehydes **108** and ammonium acetate in ethanol. In this multicomponent reaction, isoxazoles **106** react with the enolized β-ketoester **107**, to form intermediates **109** which can react with imines **110** (obtained by condensation of aldehydes **108** with NH_4_OAc) to produce the aminotrifluoromethylketones **111**. The intramolecular attack of the amino group to the highly reactive trifluoromethylketones led to the trifluoromethylpiperidines **112** (Scheme 33) [63].

#### 3.1.7. Intramolecular Nucleophilic Substitution

**Scheme 34:**
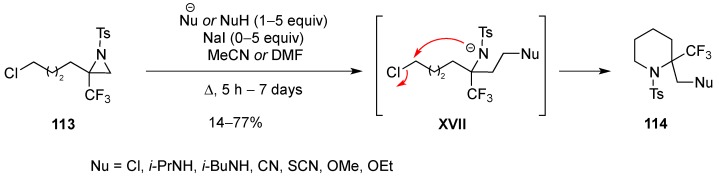
Synthesis of α-trifluoromethylpiperidines from trifluoromethylaziridines.

The intramolecular nucleophilic displacement of a good leaving group by an amine is one of the must common methods to prepare six-membered nitrogen heterocycles. This method was used to synthesize 2-trifluoromethylpiperidines **114** from the highly reactive aziridines **113**. The high reactivity of **113** is due to the presence of the trifluoromethyl group and the *N*-tosyl group. The attack of aziridine **113** by a variety of nucleophiles was very regioselective and produced intermediates **XVII** that cyclized according to an intramolecular nucleophilic substitution of the chloride to produce **114** (Scheme 34) [64].

#### 3.1.8. Intramolecular Nucleophilic Attack of Aldehydes

**Scheme 35:**
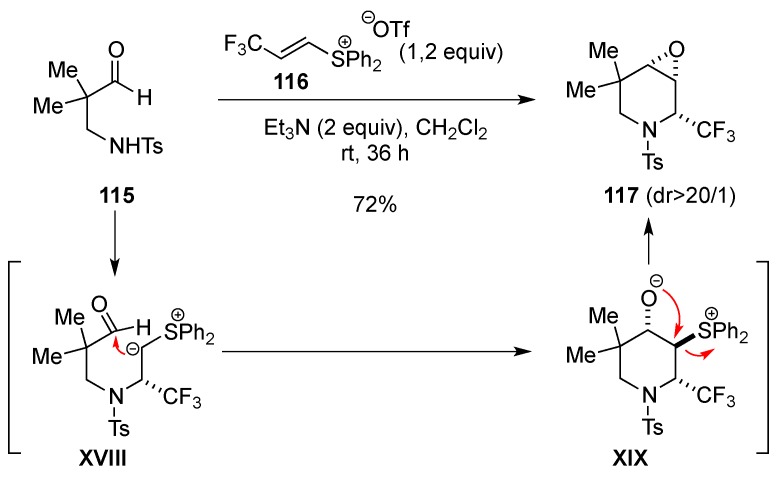
Synthesis of α-trifluoromethylpiperidino-epoxides.

The annulation of aminoaldehyde **115** was realized by an intramolecular attack of a diphenyl sulfonium species on an aldehyde. When aminoaldehyde **115** was reacted with the trifluoromethyl- vinyl diphenylsulfonium **116**, the ylide intermediate **XVIII** was formed and a cyclization took place to produce **XIX** which led to the piperidino-epoxide **117**. It is worth noting that **117** was isolated with a good yield (72%) and a good diastereoselectivity (dr > 20:1) (Scheme 35) [65].

## 4. Conclusions

Despite the considerable synthetic efforts spent to synthesize 2-trifluoromethylpiperidinic derivatives, most of these compounds have been obtained in a racemic form. When they are optically active, the methods involve a chiral auxiliary or the starting material comes from the chiral pool. Thus, there is still a strong demand for catalytic enantioselective methods to efficiently access 2-trifluoromethylpiperidine derivatives in high diastereo- and enantioselectivity.

## Figures and Tables

**Table 1:**
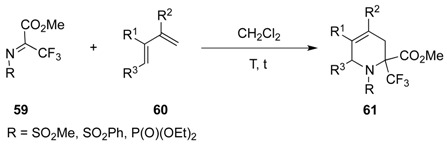
Aza Diels-Alder cycloadditions.

Entry	R	R^1^	R^2^	R^3^	T (°C)	t (h)	Yield
1	SO_2_Ph	Me	Me	H	–30 to 20	13	71%
2	SO_2_Me	Me	Me	H	–30 to 20	14	91%
3	P(O)(OEt)_2_	Me	Me	H	20	120	95%
4	SO_2_Ph	H	Me	H	–30 to 20	13	81%
5	SO_2_Me	H	Me	H	–30 to 20	13	97%
6	P(O)(OEt)_2_	H	Me	H	20	150	68%
7	SO_2_Ph	H	H	Me	4	120	75%
8	P(O)(OEt)_2_	H	H	Me	20	720	89%
